# Microstructural Evolution and Micro-Corrosion Behaviour of Flash-Welded U71Mn Joints as a Function of Post-Weld Heat Treatment

**DOI:** 10.3390/ma16155437

**Published:** 2023-08-03

**Authors:** Tingting Liao, Xi Zhang, He Yang, Pan Zhou, Fei Chen

**Affiliations:** 1 School of Materials and Environmental Engineering, Chengdu Technological University, No. 1, Section 2, Zhongxin Avenue, Pidu District, Chengdu 611730, China; 2 School of Materials Science and Engineering, Southwest Jiaotong University, Chengdu 610031, China; zxkingdom@163.com

**Keywords:** flash-butt welding, U71Mn welded joints, microstructure, corrosion behaviour

## Abstract

The microstructural evolution and corrosion behaviour of railroad flash-butt-welded U71Mn joints and the effect of heat treatment were investigated via scanning electron microscopy and electrochemical measurements. The joint structures were found to mainly comprise pearlite and a few ferrites. The grains became finer and more homogeneous after heat treatment. Additionally, there was a decrease in the corrosion current density (1.71 × 10^−5^ A cm^−2^) and increases in the absolute corrosion potential (0.86 mV) and corrosion resistance (1088.83 Ω^−1^cm^2^). This was primarily attributed to the fewer Cl^−^ ions at the homogeneous grain boundaries and fewer oxidation reactions on the joints after heat treatment. The findings of this study explain corrosion failure and will guide the development of corrosion-resistant joints for improved railroad quality.

## 1. Introduction

Since the construction of the Qinghai–Tibet railway line in China to meet the increasing demand for economic and safe railway travel, there has been a need to develop higher-quality steel rail materials to reduce rail failure and maintenance costs [1,2]. U71Mn rail steel is one of the strongest rail materials, and is widely used for rail construction [3,4]. In rail tracks, the welded joint is considered the weakest section in the seamless line, which directly affects the service life of the welded rail [5,6,7]. Among the available welding technologies, flash-butt welding is considered the most reliable for achieving stability, as demonstrated by its high efficiency, heat concentration, and high performance [8,9].

During the operation of a high-speed rail track, crack defects usually occur on the rail surface owing to manufacturing defects or long-term operational loads [10,11]. These cracks progressively result in peeling or fracturing, which limits the life span of the railway. Corrosion, which occurs via several mechanisms, is one of the main modes of failure of rail tracks [12,13,14]. Thus, understanding the corrosion characteristics of rails is important to the service life of the rail joints [15,16,17], especially in different microzones exposed to aggressive environments [18,19]. Lanzutti et al. [20] investigated Gr. 91 steel welded joints after post-weld heat treatments and developed a correlation between their corrosion and microstructure. They reported that the heat-affected zone (HAZ) is the most active site after welding and that tempering decreased its corrosion resistance. Ding et al. [21] investigated the corrosion behaviour of 316L/52M/A508 welded joints with dissimilar metals. Through electrochemical experiments, they noted the formation of a duplex structure consisting of an oxide film with different element distributions. Xu et al. [22] investigated copper/316L stainless-steel dissimilar-metal welded joints using an electrolytic copper cathode plate. They reported the formation of corrosion pits when the γ phase was dissolved and eliminated from the surface. These results were confirmed by the transformation of the galvanic material based on the unstable microstructure of the γ and ε-Cu phases. Li et al. [23] discussed the corrosion behaviour of welded aluminium alloy A7N01P-T4 joints produced for high-speed trains. The Al cladding enhanced corrosion resistance by decreasing the corrosion currents and producing positive shifts in the potentials. Fattah-Alhosseini et al. [24] studied the relationship between the microstructure and corrosion behaviour of dissimilar friction stir-welded joints and investigated the formation of equiaxed recrystallised grains.

In the case of U71Mn rail steel, which is the alloy most frequently used for railway construction in China, researchers have focused on the mechanical properties of the welded joints [25,26] instead of the corrosion performance of the steel [27,28,29]. As U71Mn welded joints are subjected to the highest risk during service, and there is a need to investigate the variations in the weld grain size with the heterogeneous chemical compositions and microstructures [26]. Additionally, there is a need to examine the corrosion performance of flash-welded U71Mn welded joints to prolong their service lives. Their electrochemical behaviour and microstructural evolution should be analysed for this purpose.

In the present study, we fabricated U71Mn welded joints via flash welding and examined the effect of a post-weld heat treatment applied at approximately 910 °C on the relationship between the joints’ microstructural evolution and corrosion performance via electron microscopy and electrochemical measurements. Our findings regarding the corrosion response mechanisms of the flash-welded U71Mn joints are expected to contribute to improving the service life and quality of steel rails and developing corrosion-resistant joints.

## 2. Materials and Methods

### 2.1. Preparation of U71Mn Welded Joints

Hot-rolled U71Mn rail sections (60 kg m^−1^) were purchased from Pan Zhihua Iron and Steel (China). Table 1 lists the chemical composition of the U71Mn rail obtained from the vacuum direct reading spectrometer (QSN750, OBLF, Dortmund, Germany) and Chinese standards [30,31] to ensure the quality of the rail before the welding technology. Welded joints were prepared using a welding machine (UN5-150ZB, Aige Technology Co., Ltd., Chengdu, China; 9350 kg, with an upsetting force of 800 kN and a rated frequency of 50 Hz). The as-produced joints are referred to as W1. Some of the welded joints were normalised in two stages using a dual-frequency induction heating machine (ZH650, Aige Technology Co., Ltd., Chengdu, China). The final temperature was set to 910 °C, and the final air blast temperature to 600 °C. The post-weld heat-treated joints are referred to as W2.

**Figure 1:**
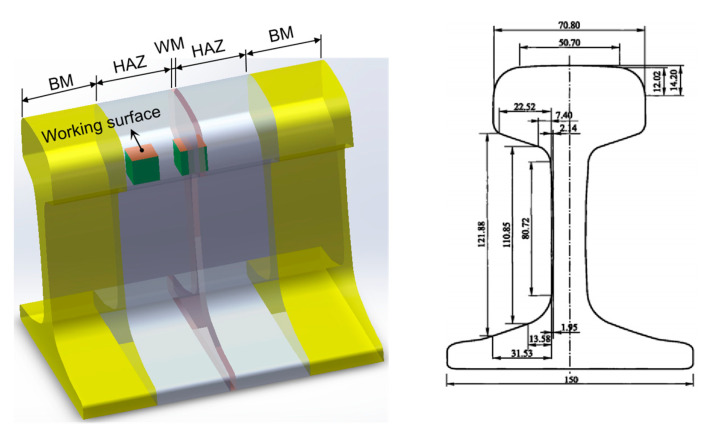
Sampling points on an U71Mn rail joint (mm).

The areas of the welded joints prepared via flash-butt welding can be divided into three sections according to their typical microstructures: the base metal (BM), HAZ, and weld metal (WM). A schematic of the sampling points of rail steel is shown in Figure 1. The working surface is the approximate size and shape of the material used for testing in each zone.

### 2.2. Electrochemical Test

To determine the electrochemical properties of each microzone of a U71Mn flash-welded joint, sample dimensions of 10 mm × 10 mm × 10 mm were obtained from the WM, HAZ, and BM, respectively. The working electrodes were connected to a copper wire and embedded into epoxy resin. Before obtaining the electrochemical measurements, the exposed areas of the working electrodes were ground using 2000-grit paper and ultrasonically cleaned with absolute ethyl alcohol. The electrochemical experiments were carried out on a CS310 electrochemical workstation, using a traditional three-electrode cell with a platinum mesh counter electrode and Ag/AgCl (saturated KCl) as the reference electrode. Before the test, the sample was immersed in a 3.5% NaCl solution for 30 min to equilibrate. The cell was then stabilised in an open circuit state by immersing it in a 3.5% NaCl solution for 30 min. To maintain a stable system, the potentio-dynamic polarisation (PDP) curve was checked at a scanning rate of 0.5 mV s^−1^ and a potential range of −500–500 mV relative to the open-circuit potential (OCP).

The corrosion potential and current density were obtained from the PDP curves using the Tafel linear extrapolation method [32]. Electrochemical impedance spectroscopy (EIS) measurements were conducted by applying an excitation voltage of 10 mV within a frequency range of 10^−2^–10^5^ Hz under the OCP condition. The EIS data were fitted using ZView software (version 3.1). To ensure repeatability, all the measurements were performed at least three times under the same conditions, including the immersion in a 3.5% NaCl solution at a temperature of 25 °C.

### 2.3. Surface Morphology

The metallographic morphologies of the U71Mn rail joints were observed using an optical microscope (AxioLab.A1, ZEISS, Jena, Germany). The morphologies of the corrosion products were investigated via scanning electron microscopy (SEM, Gemini, ZEISS, Germany). Elemental distributions were detected via energy-dispersive spectroscopy (EDS), along with SEM.

## 3. Results and Discussion

### 3.1. Metallographic Microstructural Characterisation

**Figure 2:**
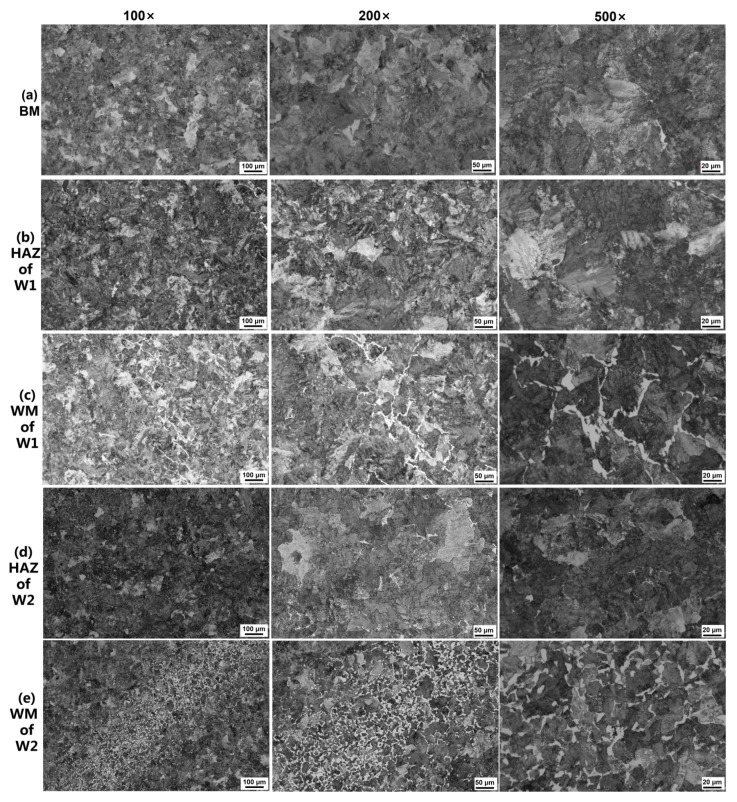
Representative optical micrographs of the metallographic microstructures of the (**a**) BM, (**b**) the HAZ of W1, (**c**) the WM of W1, (**d**) the HAZ of W2, and (**e**) the WM of W2. The scale bars (100, 50, and 20 μm) indicate the magnification (×100, ×200, and ×500, respectively).

The metallographic microstructures of the BM and welded joints are depicted in Figure 2. As shown in Figure 2a, the BM microstructure primarily consists of lamellate pearlite and a minor presence of ferrite. The BM remained unmelted during the welding process and also serves as one of the microzones of the welded joint. Figure 2b–e show the representative microstructures of different zones in the welded joints, including BM, HAZ, and WM. At low magnifications, weld seams are clearly visible in the weld zones of W1 (Figure 2c) and W2 (Figure 2e) at 100× magnification. The fusion zone is a narrow transition area (about 300 μm) between the weld metal and the base metal in the welded joint. At high magnifications (500×), reticulated ferrite encloses the pearlite in the welding line. By contrast, the size of the reticulated ferrite is about 40 μm in W1 and 20 μm in W2. Furthermore, Figure 2b,c show the coarse grain and uneven grain distributions typical of W1’s microstructure, while Figure 2d,e exhibit uniformly distributed fine grains in W2’s weld zones due to re-austenitisation after the post-weld heat treatment.

### 3.2. Polarisation Curve

**Figure 3:**
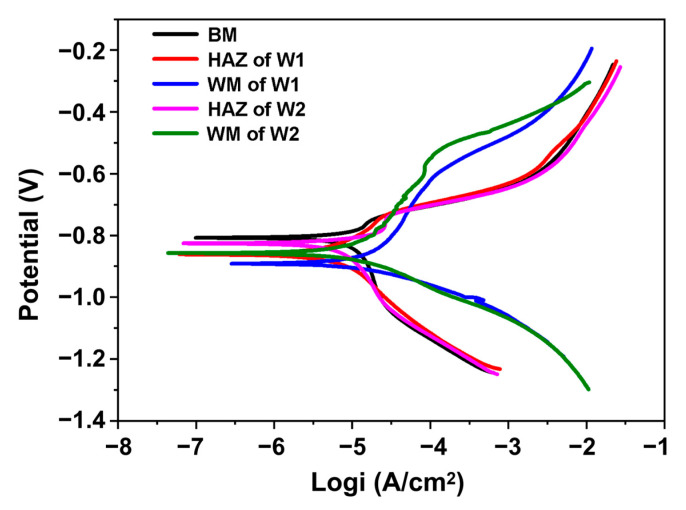
Cyclic potentio-dynamic polarisation curves of the microzones of the BM, W1, and W2.

**Figure 4:**
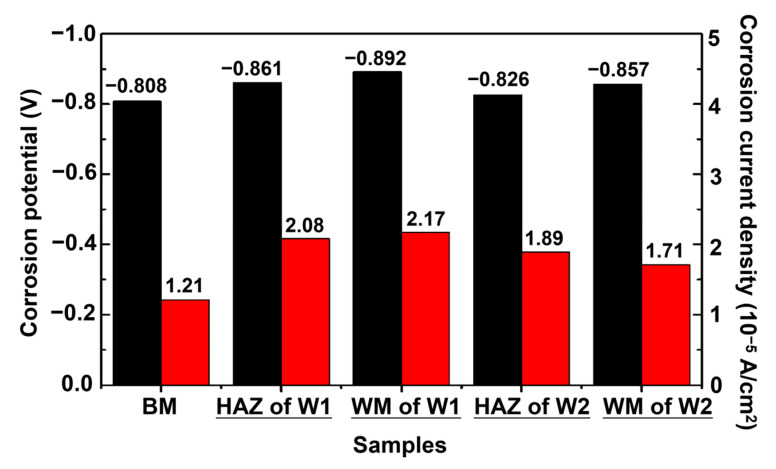
Corrosion potentials and currents of the BM and the welded joints after Tafel fitting. The black and red bars represent the corrosion currents and potentials, respectively.

Figure 3 shows the results of the potentio-dynamic polarisation tests of the BM and welded joints in a 3.5 wt.% NaCl aqueous solution. The corrosion currents and potentials obtained are summarised in Figure 4. The polarisation curves of the microzones of the welded joints are similar for both W1 and W2. Compared with the BM, the WM and HAZ exhibit more negative corrosion potentials and higher corrosion current densities, indicating higher corrosion rates. The corrosion current densities of the BM, HAZ, and WM in W2 are 1.21 × 10^−5^, 1.89 × 10^−4^, and 1.71 × 10^−4^ A cm^−2^, respectively (Figure 4). The corrosion potentials of the BM, HAZ, and WM in W2 are −0.808, −0.826, and −0.857 mV, respectively. Similar results were obtained for W1. A larger corrosion current density and larger absolute value of the corrosion potential indicate a higher corrosion rate and weaker corrosion resistance [33]. Thus, the two different microzones, WM and HAZ, exhibit higher corrosion rates and lower levels of corrosion resistance than the BM. This is attributed to the high reactivity and preferential corrosion of the WM and HAZ microzones when exposed to severe environments.

Comparing the microzones of W1 and W2, both welded joints exhibit similar corrosion potentials and current densities. The corrosion current densities of the WM of W1 and W2 were 2.17 × 10^−5^ and 1.71 × 10^−5^ A cm^−2^, respectively, and their corrosion potentials were −0.892 and −0.857 mV, respectively. Compared with W1, W2 has a lower corrosion current density and lower negative corrosion potential, indicating a lower corrosion rate and higher corrosion resistance. 

Passivation was observed in all the samples. With increasing potential, rapid anodic dissolution occurred, with the resulting passivation coating protecting the underlying metal from further corrosion. The passivation areas for the WM of W2 were larger than those of W1, indicating the higher density of the corrosion products formed on the surfaces of the welded joints of W2. This suggests that the welded joints inhibit the diffusion of the electrolyte. The potential passivation range of W2 is slightly larger than that of W1, implying a greater positive potential on the W2 surface when the passivation layer is broken. Thus, the protective ability of the passivation layer of W2 is better than that of W1.

### 3.3. Resistance

**Figure 5:**
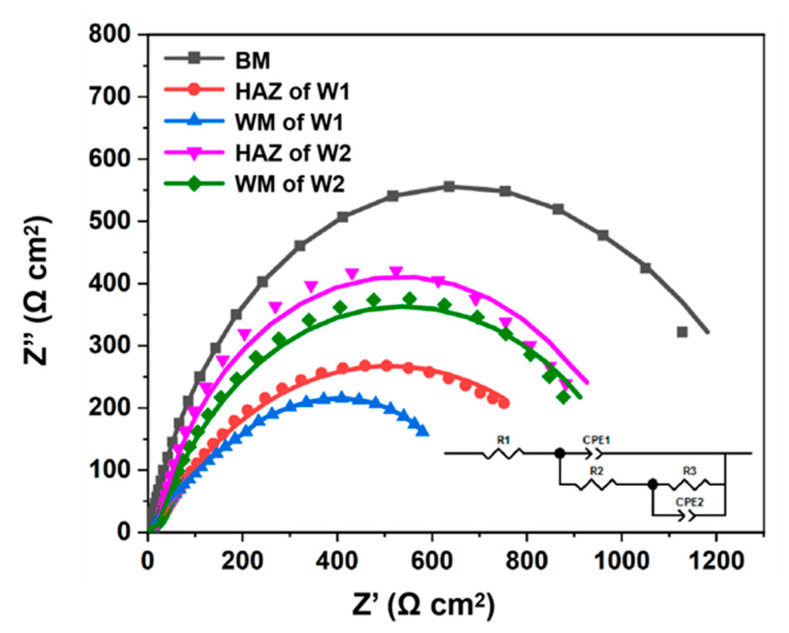
Nyquist plot of WM in a 3.5 wt.% NaCl solution. The equivalent circuit is shown in the inset.

To further investigate the EIS results, the measured data were fitted using the equivalent circuit shown in Figure 5. The Nyquist plots of the BM, W1, and W2 have capacitive loops in which the radius of the quasicircle is positively related to the resistance. The BM has the highest total resistance (R1 + R2, 1437 Ω^−1^cm^2^). In contrast, the total resistance of W2 (1056.16 Ω^−1^cm^2^) is larger than that of W1 (829.89 Ω^−1^cm^2^), suggesting a higher corrosion resistance of W2 after the post-weld heat treatment. 

**Figure 6:**
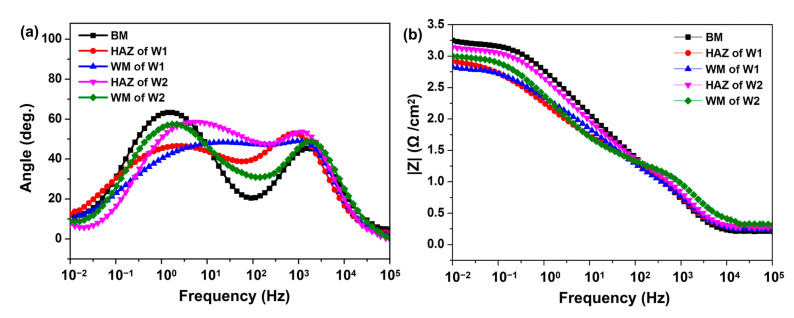
Bode plots of the U71Mn rail joints: (**a**) angle and (**b**) |Z| as functions of the frequency.

Figure 6 shows Bode plots of the U71Mn rail joints. In Figure 6a, each curve has two time-constant features within the frequency range. The maximum phase angle of the BM (~63.3°) is larger than that of the welded joints, and that of W2 (~48.8°) is slightly larger than that of W1 (~48.3°), indicating an improvement in the maximum phase angle of the welded joints after the post-weld heat treatment.

Furthermore, the value of |Z| at a fixed frequency of 0.1 Hz corresponds to the polarisation resistance, reflecting the corrosion resistance of the materials in the solution. As shown in Figure 6b, the |Z| value at 0.1 Hz for the W2 samples (2.91 Ω cm^−2^) is higher than that of W1 (2.73 Ω cm^−2^), indicating the higher corrosion resistance of W2 in a 3.5% NaCl solution.

Representative data from the Nyquist and corresponding Bode plots are shown in Table 2. Because the microstructures of the welded joints included finer and more homogeneous grains after the post-weld heat treatment, W2 exhibits a lower corrosion current density and higher corrosion resistance than W1. 

### 3.4. Morphological Analysis of the Corrosion Products

**Figure 7:**
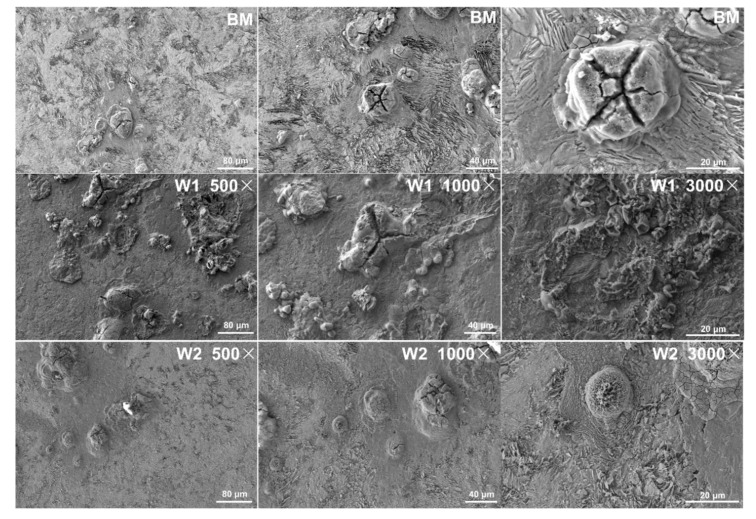
Macromorphologies of the BM and welded joints.

The corrosion morphologies of the BM and welded joints after electrochemical testing are shown in Figure 7. The BM and W2 are lightly etched and exhibit few micrometre-sized pits. Extensive localised corrosion sites are distributed in W1. Thus, mild corrosion occurred in BM and W2, whereas severe corrosion occurred in W1. These results agree well with the polarisation curve and corrosion resistance results. Irregular surfaces with cracks in the corrosion products can be observed in the BM and welded joint, suggesting the clustering of the widely dispersed corrosion pits on the surface. Compared with W2, the rust layers of W1 are more heterogeneous, with smaller spacing and more defects, indicating a lower ability of the former to protect the rail joint surfaces. This is confirmed by the occurrence of evident corrosion pits on the W1 surface.

The smaller and more uniform grains of W2 promote the formation and stabilisation of a compact and thick corrosion product layer. Ion diffusion across the grain boundaries is impeded by the thick and compact products, which suppress cathodic corrosion reactions [34]. In contrast, the focal corrosion products [35] in W1 contribute little to protecting the material owing to the large amount of internal defects and loosened structures.

**Figure 8:**
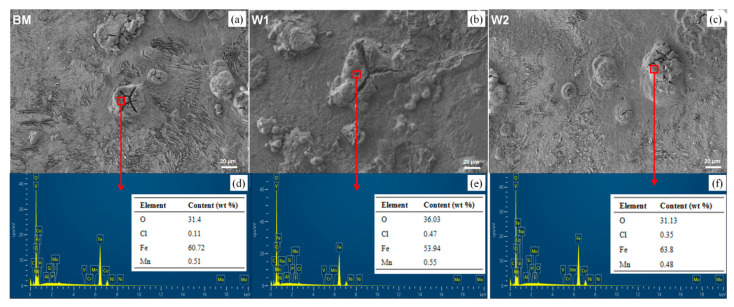
Locations selected of EDS results for the (**a**) BM, (**b**) W1, and (**c**) W2, and EDS elemental mapping of (**d**) BM, (**e**) W1, and (**f**) W2.

The elemental compositions were analysed via EDS, as shown in Figure 8 and Table 3. Compared with the corrosion-product-free sites, the corrosion products have lower Fe, Mn, and Si contents which reflect the dissolution of the metallic elements at the anodic site during corrosion. W1 has the highest Fe dissolution rate, consistent with the electrochemical results. Moreover, selective Mn dissolution in the BM and welded joints did not occur in the NaCl solution. This explains the similar rates of initial pitting of the BM and the welded joints. As a relatively stable element, Si dissolved more from the welded joints than that from the BM. However, the corrosion products were similar, illustrating the heterogeneous distributions of the metals in the local areas.

Furthermore, the Cl content originating from the corrosion solution (3.5 wt.% NaCl) is higher in the sites containing corrosion products than those without corrosion products, suggesting the adsorption of Cl^−^ on the surfaces. Interestingly, the Cl^−^ content in the corrosion products of W1 was significantly higher than in those of W2. Cl^−^-containing media contributes to an automatic operation response, resulting in more severe pitting corrosion. On the joint surface, Cl^−^ promotes the entry of the local aggregation into the exterior defect at the inhomogeneous grain boundary.

Cathodic reaction: O_2_ + 2H_2_O + 4e → 4OH^−^

Anodic reaction: 2Fe → 2Fe^2+^ + 4e

The O content in the sites with corrosion products is significantly higher than in sites without corrosion products, illustrating the oxidation reaction of the U71Mn rail joints. Compared with W2, higher O and C contents and a lower Fe content are noted in the corrosion products of W1, indicating that the Fe compound tends to form the hydroxides Fe–COOH and Fe–OOH rather than Fe_2_O_3_, FeO, and Fe–OH. Compared with the Fe oxides, Fe hydroxides are detrimental to corrosion resistance in NaCl solutions [15]. On the surfaces of the W1 welded joints, the coarse grains and heterogeneous distribution promote the unstable initiation of pits and preferential local accumulation of cathode sites, unlike in W2. For the W1 microzones, the interiors of the reaction sites are local areas of oxygen enrichment which can form stable corrosion products. Conversely, the defects of the exterior sites act as grain boundaries that form reactive sites and continue to react with Cl^−^ acceleration, thereby increasing corrosion. 

### 3.5. Corrosion Mechanism

For rail tracks in a corrosive environment, pits generally form on the surface of the rail, especially at the bottom of the rail foot [11,36]. This causes stress concentration under the superposition of internal stress and alternating axial loads, resulting in fatigue cracking [37]. Subsequently, the cracks experience sustained growth under service conditions above the crack growth threshold, which ultimately leads to the fatigue fracture of the rails [29]. The welded joints are the weakest sections of the railway owing to their heterogeneous chemical composition and microstructure [17,38]. This study focused on the corrosion mechanism of flash-butt-welded joints of U71Mn rail steel to improve the service life and quality of steel rails and develop corrosion-resistant joints. 

**Figure 9:**
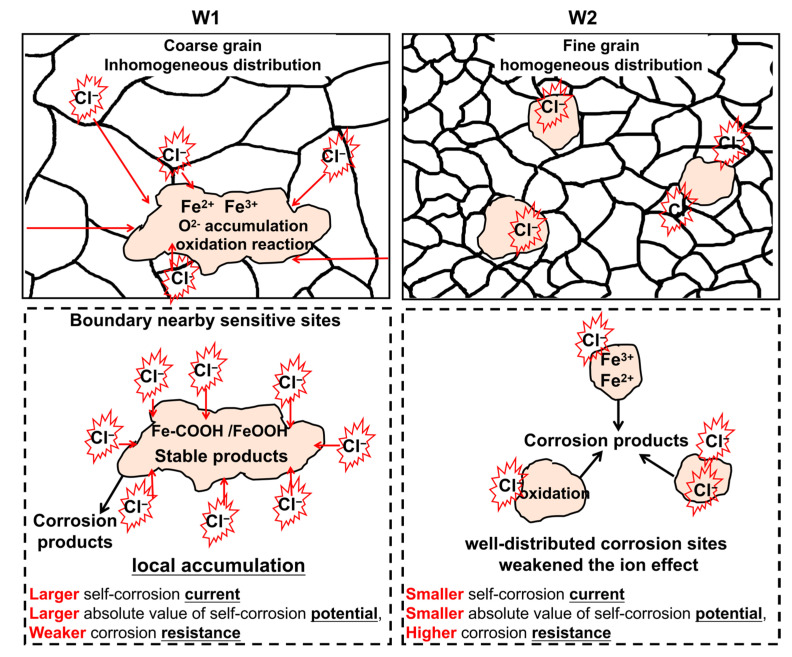
Mechanism of the corrosion response of U71Mn welded joints.

Figure 9 shows a schematic of the determined corrosion mechanisms, including the combined effects of Cl^−^ and O on the corrosion products. When these ions are adsorbed and accumulate locally, they form locally-corroded microbatteries that promote corrosion [39]. Pits generally initiate at surface inhomogeneities such as inter-metallic phases, non-metallic inclusions, grain boundaries, dislocations, defects, or mechanically damaged sites [18,40]. This readily occurs in the WM microzones of W1, with coarse grains and uneven grain distributions. In W2, well-distributed corrosion sites weaken the ion effect when exposed to the same aggressive medium. Moreover, fewer corrosion products were noted in W2 relative to W1. In addition, fewer O products and less Cl^−^ response are concentrated on W2 relative to W1. As the cladding layer has a thicker passive film which provides better corrosion protection to the cladded component [12], the products on W2 show less corrosion resistance. These results agree with the findings regarding the polarisation curve and corrosion resistance. Compared with W1, W2, with its uniform grains, has a smaller self-corrosion current (1.71 × 10^−4^ A cm^−2^) and less negative corrosion potential (−0.857 mV), indicating its lower corrosion rate and better corrosion resistance. The total resistance of W2 (1088.83 Ω^−1^cm^2^) is higher than that of W1 (811.875 Ω^−1^cm^2^), suggesting the better corrosion resistance of W2 after the post-weld heat treatment.

## 4. Conclusions

In the present study, the microstructural evolution and corrosion performance of U71Mn welded joints prepared via flash welding and subjected to post-weld heat treatments were investigated and compared with those of the BM. The microstructures of the welded joints, the polarisation curves and resistances, corrosion product morphologies, and elemental distributions were examined to elucidate the corrosion mechanism. The main findings are summarised as follows:

1. The microstructural analysis revealed that the main structure of the U71Mn welded joints and the BM consisted of pearlite and a small amount of ferrite. Due to re-austenization after post-weld heat treatment, uniformly distributed fine grains can be found in the weld zones.
2. Because the microstructures of the welded joint were disrupted and then reconfigured after welding, the U71Mn welded joints exhibited a higher corrosion current density and smaller impedance value than the BM.
3. After the post-weld heat treatment, the grain microstructure of the U71Mn weld bead was finer and more homogeneous, resulting in a smaller corrosion current density, a larger absolute self-corrosion potential value, and improved corrosion resistance.
4. When the O and Cl^−^ adsorbed and accumulated in local areas in the corrosion products of the weld microzones, these elements induced an inhomogeneous response, while a less severe corrosion response in zones after the post-weld heat treatment which were relieved with a compact and thick corrosion product layer.

## Figures and Tables

**Table 1:** Chemical composition of U71Mn rails (wt.%).

Composition	C	Si	Mn	S	P	Fe
Standard requirements [31]	0.65–0.8	0.15–0.58	0.70–1.20	≤0.025	≤0.025	Remaining
Test result	0.69	0.30	0.99	0.010	0.015	Remaining

**Table 2:** Fitting results of representative data from the Nyquist and corresponding Bode plots.

Sample	R1 (Ω)	Y_0_-Q_1_ (Ω^−1^cm^−2^S^n^)	n-Q_1_	R2 (Ω cm^2^)	Y_0_-Q_2_ (Ω^−1^cm^−2^S^n^)	n-Q_2_	R3 (Ω cm^2^)	X^2^ Chi-Square	R2 + R3 (Ω cm^2^)
BM	2.519	3.55 × 10^−5^	0.91124	18	9.92 × 10^−4^	0.86369	1419	3.42 × 10^−3^	1437
HAZ of W1	1.864	5.32 × 10^−5^	0.9378	17.64	1.59 × 10^−3^	0.6248	985.4	1.03 × 10^−4^	1003.04
WM of W1	1.753	1.85 × 10^−5^	0.9295	5.99	1.25 × 10^−3^	0.58445	823.9	1.13 × 10^−3^	829.89
HAZ of W2	2.505	4.69 × 10^−5^	0.8855	19.3	1.03 × 10^−3^	0.86046	1042	4.22 × 10^−3^	1061.3
WM of W2	2.179	3.66 × 10^−5^	0.91004	19.16	9.10 × 10^−4^	0.77121	1037	2.36 × 10^−3^	1056.16

R1, R2, and R3 are the solution, film, and double-layer resistances corresponding to the interfacial charge transfer reactions, respectively. Q represents the constant-phase elements representing the non-ideal capacitances of the electrical double layer. n < 1 indicates a frequency-dependent capacitor which shows the characteristic behaviour of an oxide film. X^2^ (chi-square) is the error of the electrical equivalent circuit fitting.

**Table 3:** Main elemental compositions of the corrosion products of the BM and the welded joints (wt.%).

Weight Percentage (wt.%)	Fe	Mn	Si	Cl	C	O
Corrosion-product-free site of BM	70.48	1.25	0.13	0.09	8.91	18.48
Products of BM	60.72	0.51	0.1	0.11	6.56	31.4
Corrosion-product-free site of W1	87.18	1.26	0.27	0.02	7.85	3.23
Products of W1	53.94	0.55	0.11	0.47	8.07	36.03
Corrosion-product-free site of W2	88.77	1.63	0.22	0.02	7.13	1.12
Products of W2	63.8	0.48	0.09	0.35	3.53	31.13

## Data Availability

The raw/processed data required to reproduce the findings of this study cannot be shared at this time because they are part of an ongoing study.
